# Storage Stability of Volatile Organic Compounds from Petrochemical Plant of China in Different Sample Bags

**DOI:** 10.1155/2020/9842569

**Published:** 2020-03-12

**Authors:** Fenglei Han, Huangrong Zhong, Ting Li, Yongqiang Wang, Fang Liu

**Affiliations:** ^1^School of Chemical Engineering, China University of Petroleum (East China), Qingdao, Shandong 266580, China; ^2^Key Laboratory of Petroleum and Petrochemical Pollution Control and Treatment, Ministry of Science and Technology, Beijing, China

## Abstract

According to the emission characteristics of volatile organic compounds (VOCs) in the petrochemical plants of China, the storage stability of VOCs for two different bags, polyester aluminum (PEA) and polyvinyl fluoride (PVF), was investigated in this study by comparing the adsorption of gas samples. A series of experiments were carried out to study the impact of different factors of sampling in the petrochemical industry. The results showed that the C2∼C3 substances can be adsorbed by the Tedlar bag, and after being refilled with pure nitrogen, the VOCs adsorbed previously by the bag material can be released. The aromatic hydrocarbon VOCs with larger molecular weight had a relatively lower recovery rate than the smaller molecular weights. And the average recovery of PEA airbags was significantly better than that of PVF airbags for storing stationary VOCs in the refinery of China. More kinds of substances can be detected in the airbags that had been added with helium protective gas, and it had a higher recovery rate for both kinds of simple bags after 24 hours of storage time, which indicated that the airbags without protective gas had adsorbed these substances.

## 1. Introduction

Recently, with the development of the urbanization, the emission of the VOCs has increased greatly [1]. The components of the VOCs are complicated and have a variety of harmfulness to the environment and the health of the people. Therefore, they had drawn public attention [2–4]. At present, researchers have focused on the study of VOCs from indoor gas, and there are few studies on VOCs emitted from refinery and petrochemistry [5, 6]. The petrochemical industry in China is the main emission source of VOCs, so the control of the VOCs in the petrochemical plant has been an urgent problem [7]. The key of the management and control is how to gather and analyze the components and the concentration of the VOCs. And most of the current researchers paid more attention on the improvement of analysis and treatment methods [8–11]. However, the collection and storage of samples, as the premise of VOCs analysis, also play an indispensable role in the accuracy of the entire analysis. The importance of VOCs storage stability is also equivalent to the analysis method of VOCs [12, 13].

Whole-air sampling and sorbent-based tubes sampling methods are the most widely used techniques for collecting gaseous VOCs under on-site or laboratory conditions [14, 15]. Among them, whole-air sampling techniques include airbag sampling and tank sampling. Each of these three methods has advantages and disadvantages. Sorbent-based tubes sampling has the advantages of small size, light weight, being easy to carry, and having multiple choices of adsorbent, which can be equipped with different targets; however, the sampling process is susceptible to human contamination; it is easy to react with the target compound to cause acquisition loss and the analysis process is complicated. The container sampling method has the advantages of quick and easy, which can collect multiple mixture samples at the same time and repeat the analysis in one sampling. However, sampling is time-consuming and labor-intensive and has high requirements on cleaning and vacuuming. It causes adsorption on high-concentration samples and is not easy to clean. The airbag sampling method is convenient and cheap for sampling and analysis, and multiple samples can be withdrawn from the bag for repetitive analysis [16], even though the compounds in the sampling bag are prone to physical leakage and chemical effects that cause the concentration to decay. In view of the complex composition of stationary source volatile organic compounds (VOCs) and the high concentration of exhaust gas in each component of the refinery, the airbag method was used for sampling and analysis in this study [17, 18].

According to the emission characteristics of VOCs in the petrochemical plants of China, the two materials airbags (PEA and PVF), most commonly used for airbag sampling, were selected for analysis. A series of research and analysis were carried out on the storage stability of VOCs, and the adsorption effect of airbags on gas samples during the experiment was studied. A series of research and analysis were carried out on the storage stability of VOCs, and the leakage loss and the adsorption of gas samples by the airbags during the experiment were studied. The purpose is to explore the differences between the two materials for the collection and storage of VOCs and to provide certain theoretical and technical support for the collection, accurate analysis, and governance research of stationary source volatile organic compounds in petrochemical enterprises.

## 2. Materials and Methods

### 2.1. Experimental Program

As VOCs collected in sampling bags were typically analyzed within a short period of time (usually less than 24–48 hours after sampling) [5, 15, 19], there were also a few experiments for analysis within three days. The stability of VOCs from stationary sources in the refinery and petrochemistry was evaluated in the sampling bags for 6 days in this study. The concentration of VOCs was analyzed at different times after sampling, at 6 h, 12 h, 18 h, and 24 h on the first day, and on the other 2nd, 3rd, 4th, 5th, and 6th days, using a gas chromatograph (Agilent 7980). The storage stability in the two kinds of bags was represented by the ratio *R*, which is shown in the following:

(1) $$\begin{array}{c} R=\frac{C_{t}}{C_{i}}\;, \end{array}$$

where *R* is the recovery rate, *C*_*t*_ stands for the concentration of “*t*” days stored in the bag (ppb), and *C*_*i*_ is the initial concentration of the VOCs (ppb). When the ratio of *C*_*i*_*/C*_*r*_ is within the range of 1*σ* precision error, that is, between 0.9 and 1.1, which means the value of VOCs is close to the initial concentration [16], and the stability can be considered as steady. Due to the large amounts of samples, the average of two parallel experiments was conducted for analysis.

### 2.2. Sampling Method

**Figure 1:**
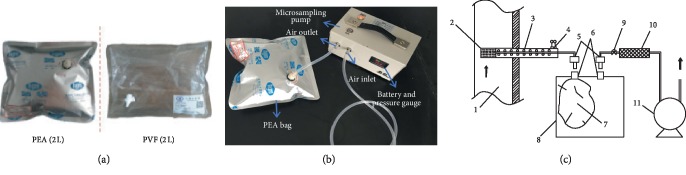
Sample airbags, the main analysis methods, and sampling devices. (a) Sample airbags. (b) Sampling instruments. (c) Sampling system. 1, exhaust pipe; 2, filter head (glass wool); 3, Teflon tubing; 4, heated sampling pipe; 5, quick connector; 6, quick connector head; 7, PVF, PEA sampling bag; 8, vacuum box; 9, valve; 10, filter (activated carbon); 11, sampling pump.

The sampling site selected the sampling hole in the prehydrogenation fractionation section of a refinery in Qingdao. And the sample airbags and sampling system are shown in Figure 1.

The sampling system was installed, and the air tightness was checked as shown in Figure 1. After the system was installed, the filter head was removed, the front end of the sampling tube was blocked, and the vacuum pressure gauge was installed on the front end of the regulating valve with a tee and connected to the Teflon tubing through the joint where the vacuum box was passed. Starting pumping gas, when the vacuum pressure gauge reached 13 kPa, the regulating valve was closed. If the vacuum pressure gauge decreased by 0.15 kPa within 1 minute, the system was supposed to have good air tightness and there was no air leakage. If the air leakage was found, a sectional inspection should be carried out to find the leakage points and causes and then repaired in time.

In order to investigate the stability of VOCs stored in the sampling bag for 6 days, sampling was performed at a fixed flow rate of 500 mL/min by using airbags 1∼4 at the sampling location (airbag 1 and airbag 2 were PEA materials and airbags 3 and 4 were PVF (Tedlar) materials). At the same time, the airbags 5∼8 (the materials of the airbag 5 and 6 were PEA, and the airbags 7 and 8 were PVF (Tedlar)) were filled with 100 mL of helium gas sampled at the same position to provide for subsequent tests. Each airbag was sent to the laboratory for analysis. Taking packaging and transportation into consideration, the volume of PEA airbag and PVF airbag was 2 L, and the volume of gas sampled was about 1.6 L per sample.

### 2.3. Instruments and Methods

The analytical instrument in this study was Agilent 7980 A. And the needle injection was used for the sample injection method and injection volume was 1 mL. The chromatographic conditions were as follows: the split ratio was 100 : 1, the postinlet temperature was 250°C, the pressure was 13.6 psi, and septum purge flow rate was 5 mL/min. The chromatographic column was Agilent 19091S-433, and the column parameters was 50 m × 200 *μ*m × 0.5 *μ*m. The initial column temperature was 35°C and increased to 300°C with 0.3 ml/min flow rate. The carrier gas was high purity helium, and the flow rate was 25 mL/min.

When examining the storage stability of VOCs for the two airbags, the entire operation cycle of the first analysis was settled as 90 minutes. After getting the material type and the substance with longest residence time, the operation cycle was changed as shown in Table 1.

## 3. Results and Discussion

### 3.1. The Stability of VOCs Stored in Two Types of Airbags

#### 3.1.1. The Adaptability of the Two Type of Airbags

**Figure 2:**
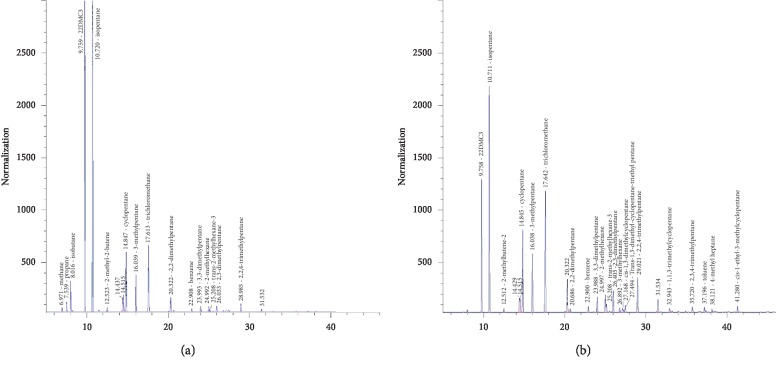
The chromatograms of 6 h on the day of sampling. (a) The PEA airbag sample chromatogram of 6 h on the day of sampling. (b) The PVF (Tedlar) airbag chromatogram of 6 h on the day of sampling.

The chromatograms of the sample stored in the two airbags for 6 hours on the sampling day are shown in the Figure 2 and Table 2. There were 16 substances that can be detected in the PEA airbags by chromatography. Meanwhile, there were 23 substances detected in the PVF (Tedlar) airbags. The above substances include alkanes, benzenes, halogenated hydrocarbons, and olefins.

**Figure 3:**
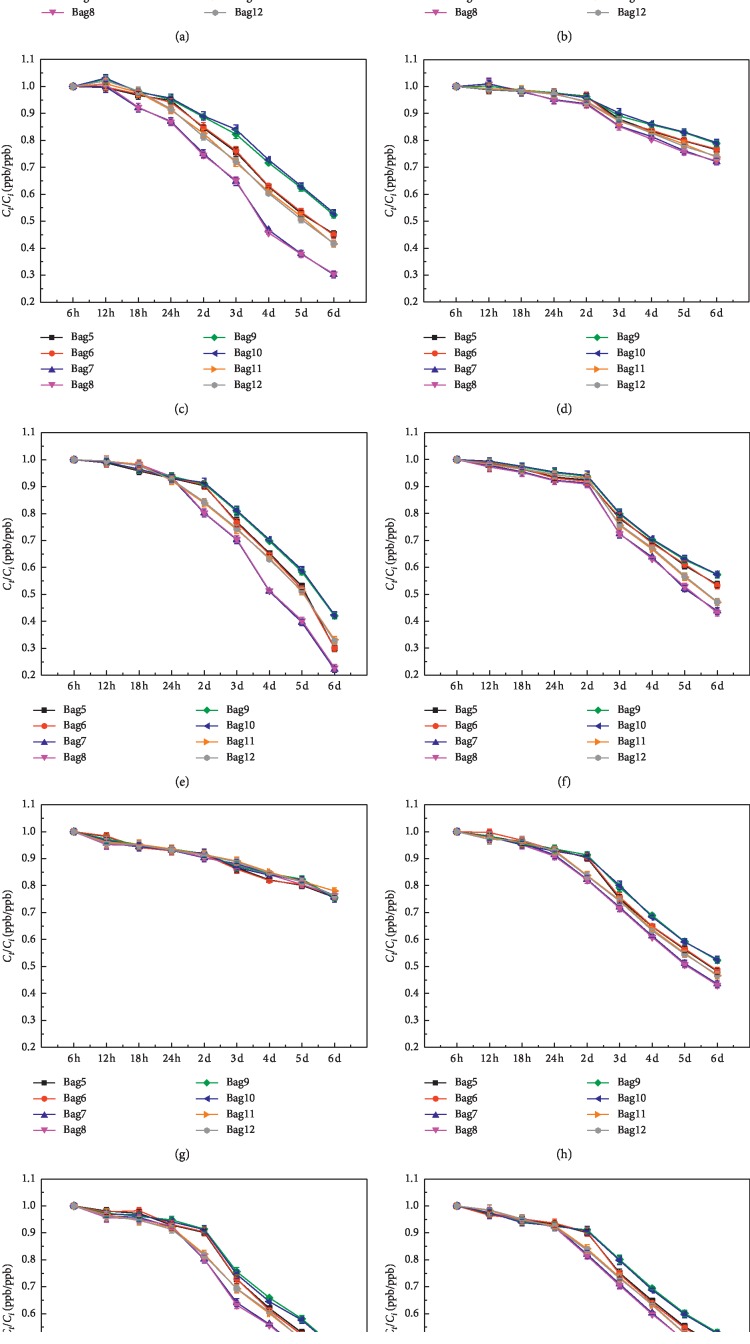
Variations of the stability of VOCs sampled in two types of airbags after 24 h. (a) 2,2DMC3. (b) Isopentane. (c) 2-Methyl-2-butene. (d) Cyclopentane. (e) Trichloromethane. (f) 3-Methylpentane. (g) Benzene. (h) 3,3-Dimethylpentane. (i) 2-Methylhexane. (j) 2,3-Dimethylpentane. *C*_*t*_ (ppb), the concentration of the target organic matter after storage for “*t*” in the airbags; *C*_*i*_ (ppb), initial concentration of target organic matter.

It can be seen clearly that more species had been detected in the PVF (Tedlar) sampling bag than the PEA sampling bag. And C1∼C3 was not detected in the PVF (Tedlar) sampling bag. It was possible that the C1∼C3 substances were adsorbed by the material of PVF (Tedlar) bag or leaked from the bag. The C5∼C8 can be detected in both airbags. After the fifth day's detection, two sampling bags were refilled with pure nitrogen and placed for 24 hours. Methane and propane were not detected in the PVF (Tedlar) airbag on the sixth day, but isobutane was detected (Figure 3). It can be inferred that methane and propane leaked through the penetration of PVF airbags, and isobutane was adsorbed on the PVF material. On the other hand, 2-methyl-2-butene, 3-methylpentane, 2-methylhexane, 2,3-two methylpentane, toluene, and other substances were detected in the PEA airbag (Figure 3). This indicated that the sample bag released volatile organic compounds adsorbed previously on the PEA bag material.

### 3.2. The Relative Recovery Rate of VOCs in PVF and PEA Airbags

14 substances that can be detected in both airbags had been chosen for studying the stability of the airbag. And the 14 substances are listed in Table 2. The storage stability of the VOCs was studied for 6 days; the histograms showed the ratio of the VOCs concentration (*C*_*t*_ (ppb)) measured on the “*t*” day (ppb) to the VOCs concentration (*C*_*i*_ (ppb)) on the first day of the initial measurement. The error bars on each histogram represent the precision error. The black dotted line in each diagram indicates the ratio of *C*_*t*_/*C*_*i*_ (ppb/ppb) of 1 : 1.

**Figure 4:**
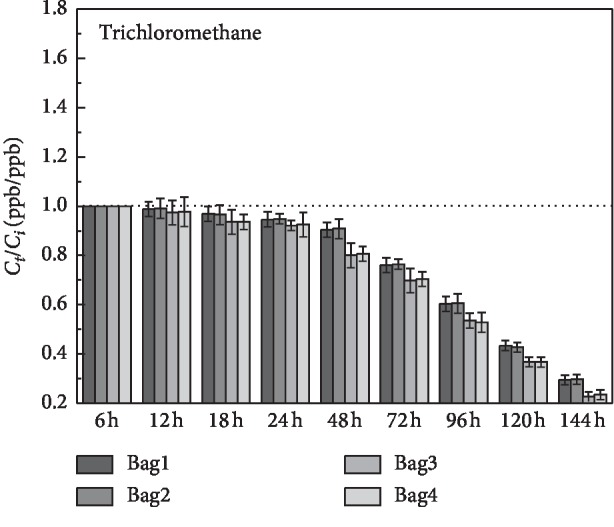
Stability analysis of trichloromethane over a 6-day storage period. Bag1/2: PEA airbag; bag3/4: PVF airbag.

It can be seen clearly in Figure 4 that halogen volatile organic compounds, trichloromethane, had more than 90% recovery rate in the first two days, and it reduced by 12% in 24 h–48 h. It had a significant decay on the third day and sustained until the sixth day. On the sixth day, the recovery rate in PVF had decreased to 23% in PVF bags and 29% in PEA bags.

**Figure 5:**
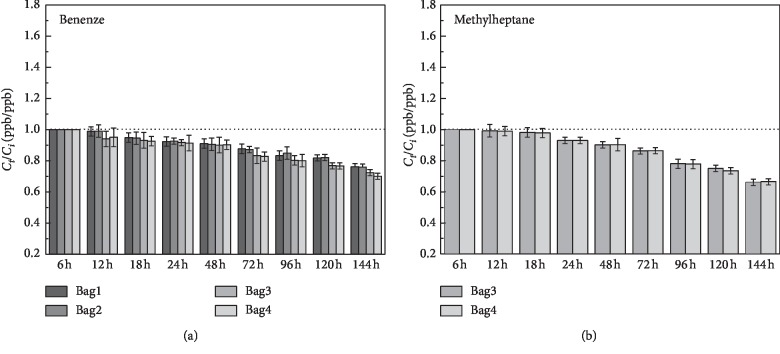
Stability analysis of benzene and methylheptane over a 6-day storage period. (a) Benzene. (b) Methylheptane. Bag1/2: PEA airbag; bag3/4: PVF airbag.

The benzene was usually used as a standard comparison substance in the chromatographic detection, and it had a higher stability in the airbags. The recovery rate of benzene in both types of airbags in the first 3 days was greater than 0.9, as shown in Figure 5(a). There was a certain degree of attenuation from the third day to the sixth day. The recovery rate in the PEA bags on the sixth day was 76% and 71% in the PVF bags. In Figure 5(b), the methylheptane was not detected in the PEA airbag. The *R* value in the first 2 days in the PVF airbag was greater than 0.9; it decreased to 0.86 on the third day and decreased to 0.66 on the sixth day. This result showed that the aromatic hydrocarbon VOCs with larger molecular weight had a relatively lower recovery rate than the smaller molecular.

**Figure 6:**
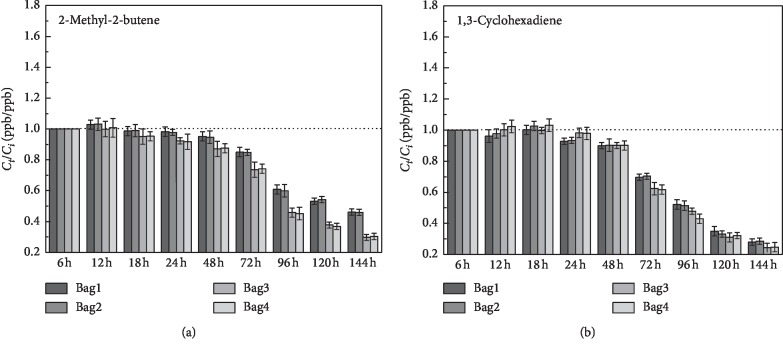
Stability analysis of 2-methyl-2-butene and 1,3-cyclohexadiene over a 6-day storage period. (a) 2-methyl-2-butene. (b) 1,3-cyclohexadiene. Bag1/2: PEA airbag; bag3/4:PVF airbag.

For the olefins, as shown in Figure 6, the recovery rates of 2-methyl-2-butene and 1,3-cyclohexadiene in the first two days of the two airbags were both greater than 90%. And it began to decrease on the third day in varying degrees. The decay rate of 1,3-cyclohexene recovery was faster than 2-methyl-2-butene, and the recovery rates of both olefins in PEA bags were greater than that in PVF bags. On the sixth day, the recovery rate of 2-methyl-2-butene in PEA was 46% (PVF: 30%), and the recovery rate of 1,3-cyclohexadiene in PEA was 28.2% (PVF: 24.5%).

**Figure 7:**
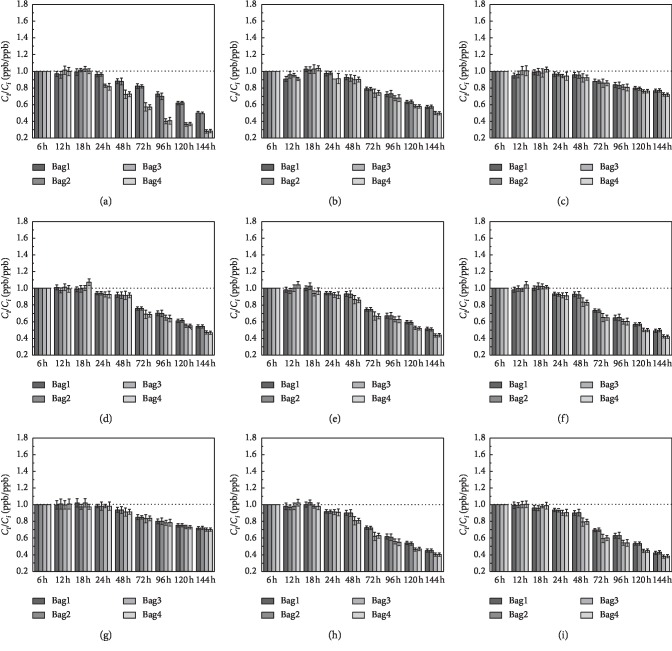
Stability analysis of captured volatile organic compounds gas over a 6-day storage period. (a) 2,2DMC3. (b) Isopentane. (c) Cyclopentane. (d) 3-Methylpentane. (e) 2,2-Dimethylpentane. (f) 3,3-Dimethylpentane. (g) Cyclohexane. (h) 2-Methylhexane. (i) 2,2,4-Trimethylpentane. Bag1/2: PEA airbag; bag3/4: PVF airbag.

The results in Figure 7 showed that, for the alkanes, the concentrations of isopentane and 2,2-dimethylpropane on the first day (*t* = 6 h) were higher than other alkanes in the same sampling bag. Between the first day (*t* = 6 h) and last day (*t* = 6 d), 9 alkanes which can be detected in both kinds of airbags as shown in Figure 7 were selected for the comparison. It can be seen clearly that PEA bags had a higher recovery rate than PVF bags for the alkanes. The recovery rate of 2,2-dimethylpropane in PEA gas bag was greater than 90% within 24 h, and in the PVF bags, it stabilized until 18 h and then began to decay. Because 2,2-dimethylpropane was a highly volatile compound, the decay increased as time went by in the airbags. On the 6th day, the recovery rates in the two airbags were 49.6% (PEA) and 28.1% (PVF), respectively. The recovery rate of cyclopentane was always the highest in 6 days in all the alkanes gases, followed by cyclohexane. The recovery rates of cyclopentane and cyclohexane in the first two days were higher than 90% in the two airbags, and there was a small attenuation on the third day, which decreased by 8.85% (PEA) and 7.3% (PVF) and 8.8% (PEA) and 8% (PVF), respectively. The recovery rate of cyclopentane and cyclohexane on the sixth day was still maintained at 76.95% (PEA) and 72.10% (PVF) and 72% (PEA) and 70% (PVF), respectively, which was much higher than the other alkanes. The recovery rates of the other alkanes had the same tendency as the cyclopentane and cyclohexane. They have recovery rates higher than 90% in the first two days, and there was a decline in different degrees from the third day to the sixth day. The recovery rates on day 6 are listed in Table 3. And it also can be seen that the recovery rate decreased with the increase of the molecular weight with the exception for the two most stable alkanes (cyclopentane and cyclohexane) and the most unstable alkanes (2,2-dimethylpropane).

The recovery rates of all VOCs during 6 days in all periods were taken into account together. It can be seen that the recovery of organic matters in all the studies had a decreasing trend with the change of storage time. The reason may be speculated that some VOCs themselves are highly active and easily decomposed and changed (e.g., 2,2-dimethylpropane). A large number of water molecules were in the gas sample, on one hand and VOCs can be indirectly adsorbed through chemical interactions (hydrogen bonding, etc.) or physical interactions (dissolution, etc.) with the water molecules. In addition, the sampling bag materials have active sites that can adsorb or catalyze VOCs.

From the overall trend, during the 6-day storage period, PEA airbags had a better storage stability for VOCs than PVF. And the average recovery rate of PEA bags (79.6 ± 4%) was better than PVF bags (72.9 ± 4%). In addition, if only the longest period of the experiment (*t* = 6 days) was compared, the average recovery rate of PEA (53.2 ± 4%) was 8.2% higher than PVF (44.6 ± 4%). The *R* values of the target VOCs in the PEA were higher than those of the PVF in all time periods analyzed in this experiment. The results showed that the storage performance of VOCs in PEA bags is better than that of PVF in the storage period of 6 days, which could promote the accuracy of VOCs analysis.

### 3.3. The Effect of Shielding Gas (Helium) on the Stability

According to Xia et al.'s research on the air sampling canister, it was found that after the inner wall of the air sampling canister was inertized, the benzene, n-hexane, and dichloromethane in the tank could be stabilized in the canister for 84 days and the concentration was basically not attenuated [20]. In this study, 100 mL of inert gas (helium gas) was introduced into the airbag before sampling to simulate the inertization of the inner wall of the Summa tank. And the airbag was stored in a dark place for 24 hours and then vacuumed and resampled to study the change trend of VOCs recovery with time.

In this study, the material of the airbags 5 and 6 was PEA, airbags 7 and 8 was PVF air, airbags 9 and 10 was PEA filled with helium gas for 24 h, and airbags 11 and 12 was PVF airbag filled with helium gas, respectively. The sample volume is 1.6 L and was taken back to the laboratory for chromatographic analysis. The analysis was carried out from the substance type, initial concentration, and stability. The results are shown in Figure 3.

**Figure 8:**
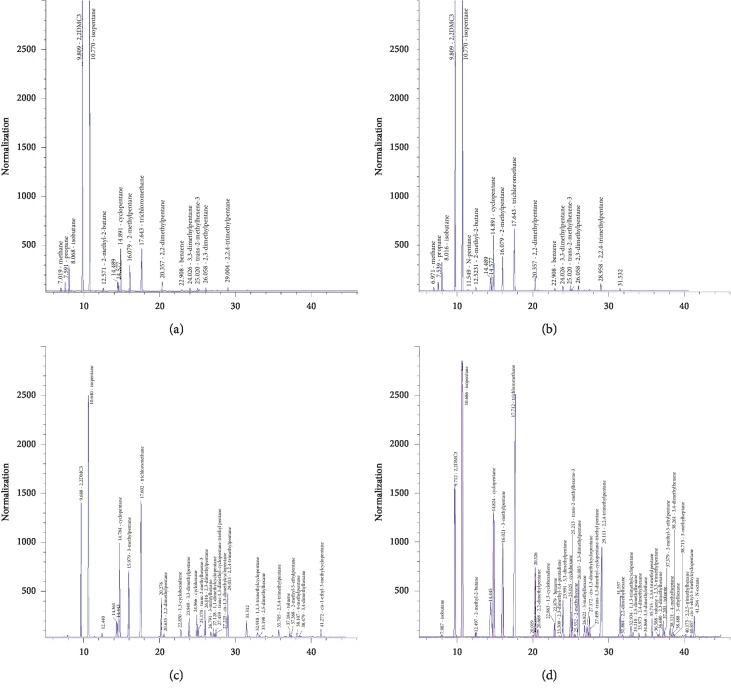
Two gas bag chromatograms of 6 h on the day of sampling. (a) PEA gas bag chromatogram without added shielding gas. (b) PEA gas bag chromatogram with protective gas. (c) PVF airbag chromatogram without protective gas. (d) PVF gas bag chromatogram with protective gas.

It can be seen clearly in Figure 8 that the kinds of substances in the PEA airbag were increased by filling with the protective gas, named n-pentane, toluene, 4-methylheptane, and an unrecognized substance with a retention time of 31.532 min, while the substances in the PVF airbag were increased by 10 kinds, with 1 isobutane, 1 C_7_H_14_ isomer, 7 C_8_H_18_ isomers, and 1 C_9_H_20_ isomer. It is shown that the airbag filled with inert gas can detect more kinds of substances after 24 hours of storage time, which indicated that the airbag without the protective gas had adsorbed these substances, and the added inert gas can replace the macromolecular substances adsorbed on the airbag material.

The basic information and peak area of the substances detected in the two airbags are listed in Table 4.

### 3.4. Stability Analysis

In this part, 10 kinds of VOCs that can be detected in both kinds of airbags were chosen for the stability analysis of the airbags. The stability of VOCs stored in airbags under two conditions was compared. The recovery rates of the two airbags filled with helium gas and the two unfilled airbags sampled after 24 hours are shown in Figure 3, in which bag5/bag6 were PEA airbags that were stored without helium condition, bag7/bag8 were PEA airbags that had been stored with helium condition, bag9/bag10 were PVF airbags that were stored without helium condition, and bag11/bag12 were PVF airbags with helium condition.

It can be seen from Figure 3 that the decay pattern of the VOCs with the addition of helium gas was similar to the VOCs that was without helium gas. Except the benzene, the stability of other VOCs in airbags was improved after 24 hours with the helium gas addition. The average recovery rate of the PEA airbag without adding helium gas after 6-day storage time was 78.43%, relatively; with adding the helium gas, the average recovery rate can be 80.93%. On the 6th day, the PEA airbag with helium added was 57.04%, which was nearly 5 percentage points higher than the unfilled recovery rate of 52.55%.

The same decay trends can be seen from the PVF gas bags. The average recovery rate for 6 days was 75.03% and 72.04% with and without helium gas added, respectively. And on the 6th day, the average recovery rate of the PVF airbag added with helium was 49.24%, which was higher than the unfilled airbags that was 44.58%. Hence, in order to ensure the varieties and concentration of the stored samples and make the storage time longer, the helium gas should be added for 24 hours before vacuuming for sampling.

## 4. Conclusion

1. Compared with PEA sampling bags, more VOCs can be detected in PVF (Tedlar) sampling bags. However, no C1∼C3 organic matter was detected in the PVF (Tedlar) sampling bags. The reason for the analysis may be that C1∼C2 small molecular substances leak and lose through permeation, and C3 may be adsorbed by the material of the PVF (Tedlar) bags.
2. Except for the two most stable alkanes, cyclopentanes and cyclohexane, and the most unstable 2,2-dimethylpropane, the recovery rate of the remaining alkanes decreased with the increase of molecular weight. The average recovery rate of all VOCs in PEA bags was better than that in PVF bags. On the 6th day, the recovery rate for the PEA bags was 8.2% higher than the PVF bags. The PEA airbags can produce more reliable data in the sampling analysis of VOCs.
3. After adding the helium protective gas, more kinds of substances can be detected. And the average recovery rate for PEA and PVF gas bags after vacuuming for 24 hours was 80.93% and 75.03%, respectively. That was higher than recovery rate without the addition of helium 78.43% and 72.04%, respectively.

## Figures and Tables

**Table 1:** Run time of the longest dwell time under different factors.

Factor	The substance with the longest residence time	Residence time (min)	Run time (min)
No protective gas	Cis-ethyl-3-methylcyclopentane	40.660	41
With protective gas	N-octane	41.294	42
Illumination effect	Cis-ethyl-3-methylcyclopentane	40.660	41

**Table 2:** The basic information and peak area of the substance were detected in the two airbags under chromatographic condition 1.

Detected substance	Residence time (min)	Peak area of the substance in the PEA airbag (PA *∗* S)	Peak area of the substance in the PVF (Tedlar) airbag (PA *∗* S)	CAS	Molecular formula
Methane^#^	6.891	184.35239	0.00000	74-82-8	CH_4_
Propane^#^	7.463	448.00043	0.00000	74-98-6	C_3_H_8_
Isobutane^#^	7.941	1487.62659	0.00000	75-28-5	C_4_H_10_
2,2DMC3^+^	9.677	2.00272*e*4	5537.92627	463-82-1	C_5_H_12_
Isopentane^+^	10.641	2.28684*e*4	9745.85938	78-78-4	C_5_H_12_
2-Methyl-2-butene^+^	12.782	213.60425	165.81610	513-35-9	C_5_H_10_
Cyclopentane^+^	14.770	3676.35522	4295.81152	287-92-3	C_5_H_10_
Trichloromethane^+^	17.642	4339.10547	4295.81152	67-66-3	CHC_l3_
3-Methylpentane^+^	15.962	2308.07642	3024.78247	96-14-0	C_6_H_14_
2,2-Dimethylpentane^+^	20.252	931.11670	162.23491	590-35-2	C_7_H_16_
1,3-Cyclohexadiene^+^	22.845	166.58859	325.45632	592-57-4	C_6_H_8_
Benzene^+^	22.900	124.57274	302.63617	71-43-2	C_6_H_6_
3,3-Dimethylpentane^+^	23.933	344.86581	821.57953	562-49-2	C_7_H_16_
Cyclohexane^+^	24.935	344.86581	152.52114	110-82-7	C_6_H_12_
Trans-2-methylhexene-3^*∗*^	25.152	0.00000	391.21350		
2-Methylhexane^+^	25.979	377.04410	934.46869	591-76-4	C_7_H_16_
2,3-Dimethylpentane^*∗*^	26.533	0.00000	1139.80676	565-59-3	C_7_H_16_
3-Methylhexane^*∗*^	26.768	0.00000	212.80467	589-34-4	C_7_H_16_
Cis-1,3-dimethylcyclopentane^*∗*^	27.250	0.00000	190.98924	2532-58-3	C_7_H_14_
1,3-1,3-Cyclopentane trimethylpentane^+^	27.446	117.51618	368.66428		
2,2,4-Trimethylpentane^+^	28.938	526.94434	1957.64331	540-84-1	C_8_H_18_
1,1,3-Trimethylcyclopentane^*∗*^	32.552	0.00000	186.32185	4516-69-2	C_8_H_16_
2,3,4-Trimethylpentane^*∗*^	35.863	0.00000	268.63855	565-75-3	C_8_H_18_
Toluene^*∗*^	37.186	0.00000	249.94514	108-88-3	C_7_H_8_
4-Methylheptane^*∗*^	38.025	0.00000	162.82713	589-53-7	C_8_H_18_
Cis-1-ethyl-3-methylcyclopentane^*∗*^	40.660	0.00000	306.85803		

^*∗*^Substance representatives can only be detected in PVF. ^#^Substance representatives can only be detected in PEA. ^+^Substance represented can be detected in both airbags.

**Table 3:** The VOCs recovery rates in 6 days.

VOCs	*R* (*t* = 6 h)		*R* (*t* = 24 h)		*R* (*t* = 48 h)		*R* (*t* = 3 d)		*R* (*t* = 6 d)	
	PEA	PVF	PEA	PVF	PEA	PVF	PEA	PVF	PEA	PVF
Trichloromethane	1	1	0.947	0.923	0.906	0.803	0.762	0.701	0.295	0.230
Benzene	1	1	0.958	0.933	0.917	0.902	0.871	0.830	0.761	0.710
Toluene	1	1	0.931		0.902		0.863		0.662	
2-Methyl-2-butene	1	1	0.980	0.920	0.949	0.873	0.849	0.739	0.460	0.300
1,3-Cyclohexadiene	1	1	0.932	0.981	0.901	0.805	0.700	0.62	0.282	0.245
2,2-Dimethylpropane	1	1	0.962	0.820	0.879	0.724	0.821	0.571	0.496	0.281
Isopentane	1	1	0.976	0.910	0.924	0.900	0.789	0.740	0.574	0.499
Cyclopentane	1	1	0.965	0.943	0.957	0.922	0.876	0.858	0.769	0.72
3-Methylpentane	1	1	0.941	0.925	0.92	0.916	0.757	0.681	0.544	0.469
2,2-Dimethylpentane	1	1	0.941	0.918	0.930	0.861	0.747	0.666	0.510	0.437
3,3-Dimethylpentane	1	1	0.928	0.911	0.925	0.831	0.733	0.649	0.495	0.423
Cyclohexane	1	1	0.982	0.981	0.936	0.913	0.849	0.834	0.719	0.7
2-Methylhexane	1	1	0.918	0.91	0.901	0.808	0.723	0.624	0.45	0.400
2,2,4-Trimethylpentane	1	1	0.910	0.901	0.902	0.793	0.697	0.597	0.425	0.380
Average/SD									0.532	0.446

**Table 4:** The basic information and peak area of the substance were detected in the two airbags under chromatographic condition 1.

The detected substances	Dwell time (min)	Peak area of the detected substance in the PEA bag (PA *∗* S)	Peak area of the detected substance in the PEA bag with protective gas (PA *∗* S)	Peak area of the detected substance in the Tedlar airbag (PA *∗* S)	Peak area of the detected substance in the Tedlar bag with protective gas (PA *∗* S)
Methane	6.891	160.51048	206.27383	0.00000	
Propane	7.463	399.86758	503.97580	0.00000	
Isobutane	7.941	1276.13147	1618.72913	0.00000	107.64230
2,2DMC3	9.677	17007.04632	21177.96536	6039.83643	6666.43945
Isopentane	10.641	19053.12843	23114.32197	11142.20184	17543.92419
2-Methyl-2-butene	12.782	182.71281	222.55095	190.42331	239.14281
Cyclopentane	14.770	3083.74121	1689.47852	5281.02295	5127.13770
Trichloromethane	17.642	2077.10547	2495.81152	6722.04772	12849.24814
3-Methylpentane	15.962	1914.48938	2206.66724	3750.27563	5601.77539
2,2-Dimethylpentane	20.252	0.00000	0.00000	209.09560	590.69238
1,3-Cyclohexadiene	22.845	0.00000	0.00000	444.73907	557.96204
Benzene	22.900	138.96864	142.95226	463.34153	657.10657
3,3-Dimethylpentane	23.933	274.80710	303.47922	1118.84204	3151.50928
Cyclohexane	24.935	0.00000	0.00000	0.00000	2288.374
Trans-2-methylhexene-3	25.152	108.87604	120.37717	522.65619	1453.50232
2-Methylhexane	25.979	258.88705	282.16379	1279.25708	115.08427
2,3-Dimethylpentane	26.533	297.42404	324.19095	1560.00342	4424.88916
3-Methylhexane	26.768	0.00000	0.00000	296.01270	834.05493
1,1-Dimethylcyclopentane	27.162	0.00000	0.00000	266.75485	402.61215
Cis-1,3-dimethylcyclopentane	27.250	0.00000	0.00000	129.11159	665.96222
Anti-1,2-dimethylcyclopentane	27.494	0.00000	0.00000	0.00000	811.83246
Trans-1,3-dimethyl-cyclopentane-triethyl pentane	27.446	0.00000	0.00000	512.21930	640.01367
2,2,4-Trimethylpentane	28.938	0.00000	0.00000	2812.03467	8030.94824
2,2-Dimethylhexane	31.864	0.00000	0.00000	0.00000	129.75447
1,1,3-Trimethylcyclopentane	32.552	0.00000	0.00000	266.71378	762.00024
2,5-Dimethylhexane	33.207	0.00000	0.00000	104.56830	299.98367
2,4-Dimethylhexane	33.973	0.00000	0.00000	0.00000	242.29050
3,3-Dimethylethane	34.870	0.00000	0.00000	0.00000	262.06342
2,3,4-Trimethylpentane	35.863	0.00000	0.00000	417.08096	1179.48853
2,3-Dimethylhexane	36.680	0.00000	0.00000	0.00000	174.69110
Toluene	37.186	0.00000	0.00000	347.34723	1015.58044
2-Methyl-3-ethylpentane	37.377	0.00000	0.00000	102.96656	298.92148
4-Methylheptane	38.025	0.00000	0.00000	225.72748	661.07220
3,4-Dimethylhexane	38.261	0.00000	0.00000	109.72504	107.13146
3-Ethylhexane	38.488	0.00000	0.00000	0.00000	319.67789
3-Methylheptane	38.713	0.00000	0.00000	0.00000	121.65337
2,2,5-Trimethylhexane	40.175	0.00000	0.00000	0.00000	179.53453
Cis-ethyl-3-methylcyclopentane	40.660	0.00000	0.00000	422.31396	132.12921
N-octane	41.294	0.00000	0.00000	0.00000	1275.79590
Total amount		5.99532*e*4		4.10868*e*4	

## Data Availability

The data used to support the findings of this study are available from the corresponding author upon request.
